# In Silico Analysis of SARS-CoV-2 Non-Structural Proteins Reveals an Interaction with the Host’s Heat Shock Proteins That May Contribute to Viral Replications and Development

**DOI:** 10.3390/cimb45120638

**Published:** 2023-12-18

**Authors:** Mthembu Yamkela, Zingisa Sitobo, Xolani H. Makhoba

**Affiliations:** 1 Department of Microbiology and Biochemistry, University of Fort Hare, Alice Campus, Alice 5700, South Africa; 201820224@ufh.ac.za (M.Y.); 201612748@ufh.ac.za (Z.S.); 2 Department of Life and Consumer Sciences, College of Agriculture and Environmental Sciences, University of South Africa (UNISA), Florida Campus, Roodepoort 1709, South Africa

**Keywords:** SARS-CoV-2, NSP2, polyamines, heat shock proteins, COVID-19, AdoMetDC, docking, molecular dynamics, pharmacophore

## Abstract

The non-structural protein 2 (NSP2) is an RNA-binding protein involved in coronavirus genome replication, and it often decreases human immune response to promote viral invasion and development. It is believed that the NSP2 associates itself with polyamines and heat shock proteins inside the host cell to proceed with viral development. This study aimed to investigate how the SARS-CoV-2 virus’ key non-structural proteins (NSP2) utilize polyamines and heat shock proteins using a molecular docking approach and molecular dynamics (MD). ClusPro and HADDOCK servers were used for the docking and Discovery Studio, chimera, and PyMOL were used for analysis. Docking of the heat shock proteins 40 (HSP40), 70 (HSP70), and 90 (HSP90) with SARS-CoV-2 NSP2 resulted in 32, 28, and 19 interactions, respectively. Molecular dynamics revealed Arg458, Asn508, Met297, Arg301, and Trp417 as active residues, and pharmacophore modeling indicated ZINC395648, ZINC01150525, and ZINC85324008 from the zinc database as possible inhibitors for this NSP2.

## 1. Introduction

At the end of 2019, China reported numerous cases of patients with pneumonia of no known cause to the World Health Organization (WHO). The virus was later identified as severe acute respiratory syndrome coronavirus 2 (SARS-CoV-2). Because so many patients were infected with the virus in a short amount of time, an outbreak of coronavirus-associated acute respiratory sickness was declared, and it was given the designation coronavirus disease 19 (COVID-19). According to the World Health Organization (WHO)’s reports, there were millions of confirmed cases of COVID-19, which resulted in hundreds of thousands of deaths worldwide. To date, no treatment has been found for this disease, and hence, it remains a threat [1]. This epidemic forced the closure of numerous businesses, both large and small. As a result, thousands of individuals lost their jobs and income. COVID-19 also caused an enormous economic shock to the international economy, with a 3.5% decrease in global production in 2020, affecting all regions of the world, regardless of their size, wealth, or poverty [1].

The SARS-CoV-2 virus is a positive-sense RNA virus that is generally identified by the size of its genome (30 kb). It also contains a 5′ cap structure and a 3′ tail, allowing this genome to exist as an mRNA that is utilized to translate polyproteins which are viral replicates [2]. It can also be identified by the club-like features on the surfaces, known as spikes, and its unique replication method. In the cell’s cytosol, the virus functions as mRNA and is translated by the host cell’s protein-making machinery, resulting in the production of important enzymes known as non-structural proteins (NSP), each of which serves a particular purpose in viral growth. Sixteen of these non-structural proteins are produced. Most of these have been studied while a few are yet to be understood with respect to their functions, conformation, and association. To further the understanding of the function of NSPs in SARS-CoV-2, this study focused on the less studied NSP2. NSP2 has been implicated in host-immune evasion during SARS-CoV-2 viral invasion, although this remains to be confirmed [3]. A previous study by Cornillez-Ty and colleagues reported that NSP2 interacts with prohibitin 1 (PHB1) and PHB2 and may be involved in the disruption of intracellular host signaling during SARS-CoV infections [4].

The SARS-CoV-2 virus might hijack the host’s biological processes, including the functioning of molecular chaperones called heat shock proteins (HSPs). These HSPs are highly associated with the protection of the cell against various stresses and are often referred to as housekeepers, acting as a maintenance machinery that helps in proteostasis, thereby preventing cell death. They differ in molecular size and are classified on the basis of these sizes [5]. The heat shock protein 40 family, known as DnaJ in prokaryotes, are co-chaperones and consist of many distinct members. These are categorized by the presence of a conserved J domain in their structure made up of approximately 70 residues and this is used to stimulate HSP70 [5,6]. According to [6], HSP40 coordinates the activation of HSP70 ATPase with substrate delivery to HSP70.

HSP70 is a molecular chaperone that is made up of the N-terminal domain, and the C-terminal domain which is responsible for substrate binding. Upon activation by HSP40, Hsp70 binds with an unfolded substrate and partially folds it. HSP70 with the partially folded substrate then binds with HSP90, another chaperone molecule which is vital in completing the protein-folding process [7]. The binding of these two proteins is facilitated by the HSP70-HSP90 organizing protein (Hop) [8]. The Hop is a highly ubiquitous nuclear and cytosolic protein. The Hop protein is expressed at moderate levels in most organisms under normal conditions and overexpressed under stressful conditions [8].

Other interesting molecules that play important roles in living organisms are molecules known as polyamines. According to Mattei et al. (1999), polyamines are biological molecules carrying a positive charge, and these include spermine, spermidine, and putrescine. These molecules are said to be important factors because they play a huge role in major bodily processes including growth, proliferation, and survival [9]. Due to the abundance of polyamines within the cells and their relevance in nucleotide charge neutralization, among other tasks, it is not a surprise that viruses exploit and modify polyamines for their replication [7]. Polyamines are required by viruses at multiple stages of their life cycle, including genome packing, DNA-dependent RNA polymerization, genome replication, and viral protein translation. These polyamines are synthesized differently from species to species, and, most importantly, their biosynthesis in mammals, including humans, is regulated by two enzymes called adenosylmethionine decarboxylase 1 and ornithine decarboxylase. The enzyme adenosylmethionine decarboxylase (EC 4.1.1.50) catalyzes the conversion of S-adenosylmethionine to S-adenosylmethioninamine. AdoMetDC (S-adenosylmethionine decarboxylase) is a key regulator in the polyamine biosynthetic pathway because it creates the n-propylamine residue necessary to produce spermidine and spermine from putrescine. It is expressed as an inactive proenzyme that auto-processes into an active enzyme. An internal serinolysis reaction leads to the cleavage of the backbone into subunits, with the latter being the smallest subunit, and the production of a pyruvoyl group at the chain’s N-terminus [10]. Bioinformatics have been widely used in drug discoveries and provide a simple starting point for novel drug production.

## 2. Materials and Methods

### 2.1. Sequence Attainment and Analysis

The sequences were obtained from the NCBI website (https://www.ncbi.nlm.nih.gov) for SARS-CoV-2 NSP2 (accession number: P0C6U8) and human heat shock proteins, heat shock protein 40 ((HSP40) accession number: O7595340), heat shock protein 70 ((HSP 70/HSPA4) accession number: P34932 70), heat shock protein 90 ((HSP 90) accession number: P08238), and adenosylmethionine decarboxylase 1 (AdoMetDC) AAH00171.1.

#### The 3D Structural Modeling and Validation

The Phyre2 (http://www.sbg.bio.ic.ac.uk) [11] database generates the 3D structures and compares the homologs found in the proteins of different species, including the structure, function, and mutations. The Phyer2 was selected because it uses advanced docking algorithms providing 3D model structures for human HSP40, HSP70, and HSP90 which were downloaded in PDB format. This server allows you to paste a Fasta sequence and use its inbuilt parameters to model the submitted sequence, no parameters are set manually. The modeled structures were visualized with PyMol v1.8 [12], Chimera v1.17.3 [13], and Discovery Studio v4.5 The AdoMetDC structure was also generated using Phyre2 together with the SARS-CoV-2 NSP2 structure. For further validation, the ERRAT website (https://saves.mbi.ucla.edu/) was used as it allows the submission of the refined protein model and graphically provides results with overall quality factors in percentages.

### 2.2. Molecular Docking and Analysis

For protein–protein docking, HSP40, HSP70, and HSP90 were docked with SARS-CoV-2 NSP2 using ClusPro (https://bioinfo3d.cs.tau.ac.il) [14,15]. ClusPro allows the input of two modelled structures, the receptor and the ligand, and no parameters are set manually. Analysis was performed through visualization using PyMOL v1.8 [12] and Discovery Studio v4.5. For the investigation of possible interactions between SARS-CoV-2 NSP2 and polyamines, the enzyme AdoMetDC was used due to the important role it plays in the polyamine biosynthesis pathway.

### 2.3. Protein–Protein Docking

Protein–protein docking was studied to understand better the dynamics between two protein molecules. HADDOCK v2.4 [16] was used for the docking analysis. This server was selected because it uses experimental or bioinformatic-derived data (ambiguity restraints) to drive the docking process. These data include NMR-derived intermolecular distances, residual dipolar couplings, or site-specific mutagenesis data, which help improve the accuracy of the predictions. Both proteins’ (HSP70 and HSP90) active residues were identified using information on the protein data bank and structural data (Protein Data Bank, https://www.rcsb.org/). The conformational space of the protein–protein complex was sampled by executing the docking in unbound, rigid-body, and flexible refinement phases. The HADDOCK v2.4 server was programmed to generate 1000 dock poses; the top two hundred were chosen for further improvement. The docked complexes were analyzed using LigPlot++ (v1.4.2), a tool designed to visualize protein–ligand interactions [17] and their binding affinities were analyzed using the AREA AFFINITY server (https://affinity.cuhk.edu.cn/index accessed on 17 May 2023). This server allows for the pasting of the docked proteins and no parameters were set on the server. Molecular dynamic simulations were conducted to evaluate the stability of the docked complexes.

### 2.4. MD Simulations

The complexes were each placed in a dodecahedron-shaped water box containing TIP3P water molecules, sodium, and chloride ions to imitate an in vivo environment. An energy-efficient steepest descent was applied for 200 ps, followed by thermodynamic equilibrium using the CHARMM36 force field and GROMACS v4.5 to ensure the system was stable. A 100 ns molecular dynamics simulation was then undertaken, with a long-range Van der Waals cut-off rvdw set at the energy minimization stage [18]. The gen_vel option was activated to commence the simulation with a gen_temp of 310 K and −1 gen seed. After the heating stage, the system was equilibrated for 15 ns, followed by another 10 ns of a relaxed MD run. During the final molecular production dynamics step, tau p was set to 2.0, and ref p was set to 1.0, utilizing the Parrinello–Rahman method for pressure coupling. Several tools, including PyMOL (v2.4) and VMD (v1.9.3), were utilized to calculate hydrogen bonding and assess the simulation findings. Analysis was performed using the GROMACS (v4.5) inbuilt modules such as gmx rms, gmx rmsf, gmx area, and gyrate [19]. mmPBSA analysis was performed, and a residue-wise heatmap was generated to study the binding interaction.

### 2.5. Pharmacophore Modeling

Structure-based pharmacophore models were generated with ZINCPharmer (http://zincpharmer.csb.pitt.edu/ accessed on 14 July 2023) [20]. Interactions between ligands and proteins were automatically analyzed considering both electrostatic and geometric (distances, size, and angles) properties of the binding regions. The generated conformers were converted into an efficient search format using the Pharmer (zincpharmer.csb.pitt.edu, accessed on 14 July 2023) [21] open-source software. Pharmer identifies hydrophobic, hydrogen bond donor/acceptor, positive/negative ions, and aromatic pharmacophore features using the SMARTS matching functionality of the OpenBabel toolkit v2.4.1 [22]. The screened molecules were then docked using autodock vina v1.2.0.

## 3. Results

### 3.1. Sequence Retrieval and Analysis

In this study, we used three heat shock proteins selected due to their importance in the human cell and the overall biological processes, and these are human heat shock proteins (HSPs) 40 kDa, 70 kDa, and 90 kDa (Table 1). Their information (FASTA sequences) was attained from the NCBI website and was analyzed using the ensemble website. The proteins used were from the human organism source as this study aims to provide a clear insight into how these proteins interact inside the human cell to fight against foreign substances. According to the analysis of these sequences, these three proteins are localized mainly in the cytosol (see Table 1), which makes it much easier for HSP40 to interact with its partner protein which is HSP70. Inside the cell, these proteins are mainly found in different locations regarding chromosomes and have a different number of exons and introns (for the number of each protein refer to Table 1).

According to Sakharkar et al. (2004), protein-coding sequences are divided into smaller sections of coding sequences known as exons, which are separated by non-coding regions known as introns. Those exons and introns are included in the first messenger RNA products when genes are transcribed, but they do not interfere with the functioning or continuous cellular interactions of these proteins. However, introns are deleted during the splicing process, leaving just exons in the final mRNA, which are utilized to determine which proteins are to be generated [23].

The structural analysis of these proteins was further performed using the NCBI conserved domain (CD) website to observe the conserved domains in each protein. Sequence analysis of human heat shock protein 40 (HSP40) revealed that the J domain is made from amino acids at position 1–343; this domain is a functional domain for this protein, and hence it should be noted. The nucleotide-binding domain (NBD) of HSP70 is composed of amino acids from positions 2–384, and the substrate-binding domain (SBD) is composed of amino acids from positions 499–743. The HSP90 chaperon has an N-terminal nucleotide-binding domain (NBD) at position 191–712 and a substrate-binding domain (SBD) at position 23–209. The above protein conservatory information obtained was then verified using the alignment to see whether these crucial domains are conserved on these proteins across different species.

### 3.2. Three-Dimensional Homology Modeling and Validation

**Figure 1:**
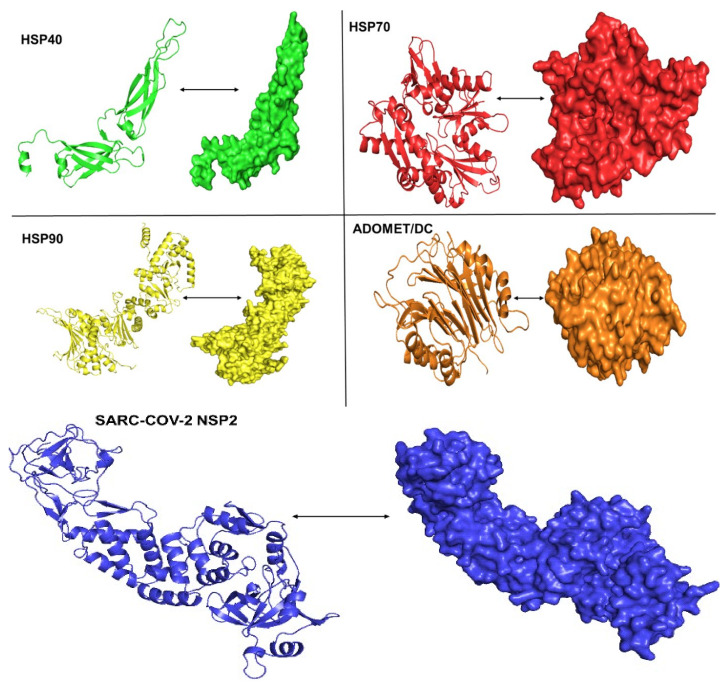
A 3D model showing beta sheets and alpha helices and surfaced structures for the human heat shock proteins 40, 70, and 90, the 3D structure of the polyamine regulating enzyme AdoMetDC, and 3D modeled structure for the SARS-CoV-2 NSP2.

The Phyre2 software program was used to refine the structures of HSP40, 70, and 90, the polyamine AdoMetDC, and the viral protein NSP2. The structural models obtained are shown in Figure 1. These models were selected because they exhibited a 100% confidence level of modeling and had an over 85% identity score compared to the originally submitted sequences.

**Figure 2:**
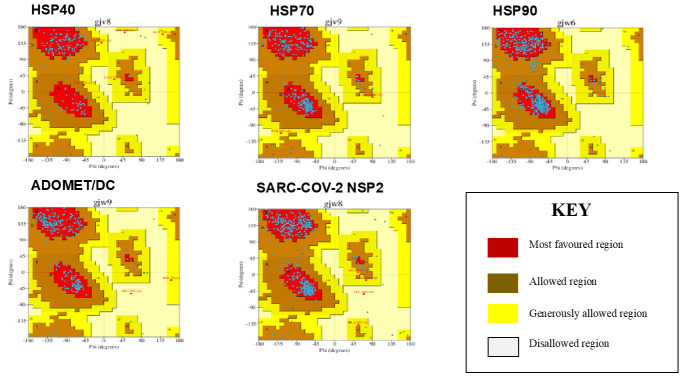
Ramachandran plots of the modeled structures shown HSP40, HSP70, HSP90, AdoMetDC, and SARS-CoV-2 NSP2. A/a represents alpha helices, B/b represents beta sheets at different plots regions, and blue dots represent amino acids.

The models were validated using Ramachandran plots (Figure 2) in the ‘PROCHECK’ tool (v3.5.4), which identifies amino acid percentages. According to PROCHECK, the percentage of residues in the most favored area should be 90% for a good quality model and superior stereochemical properties in the core region of Ramachandran plots. These Ramachandran plots depict the most favored, additionally allowed, generously allowed, and disallowed area residues in red, yellow, light yellow, and white, respectively.

The percentages of residues in the most preferred area for HSP40 (accession number: O75953), was 89.6% in the most favored region, 6.9% in the additionally allowed region, 2.8% in the generously allowed region, and only 0.2% in the disallowed region. HSP70 (accession number P34932), was 92.4% in the most favored region, 7.0% in the additionally allowed region, 0.6% in the generously allowed region, and 0% in the disallowed region. HSP90 (accession number: P08238) was 89.1%, in the most favored region, and 10.9% in the additionally allowed region. AdoMetDC (accession number: AAH00171.1) was 90.1% in the most favored region, 0.6% in the additionally allowed region, 0.6% in the generously allowed region, and 0.2% in the disallowed region. NSP2 (accession number: P0C6U8), was 90.3% in the most favored region, 9.2% in the additionally allowed region, 0.3% in the generously allowed region, and only 0.3% in the disallowed region.

**Figure 3:**
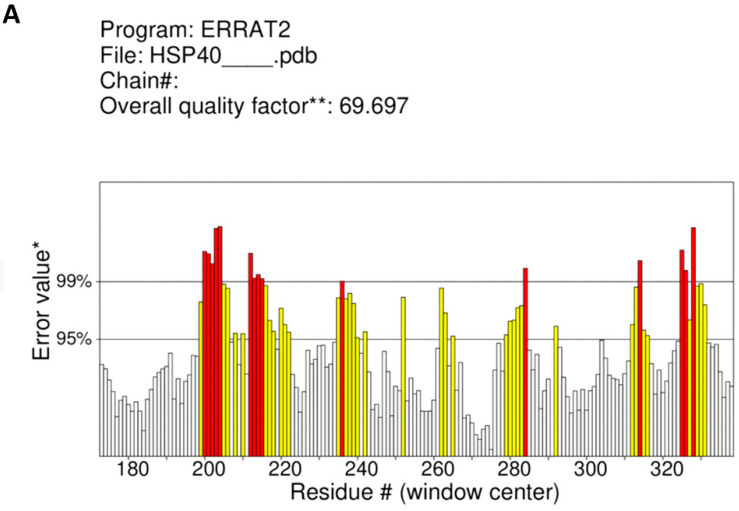

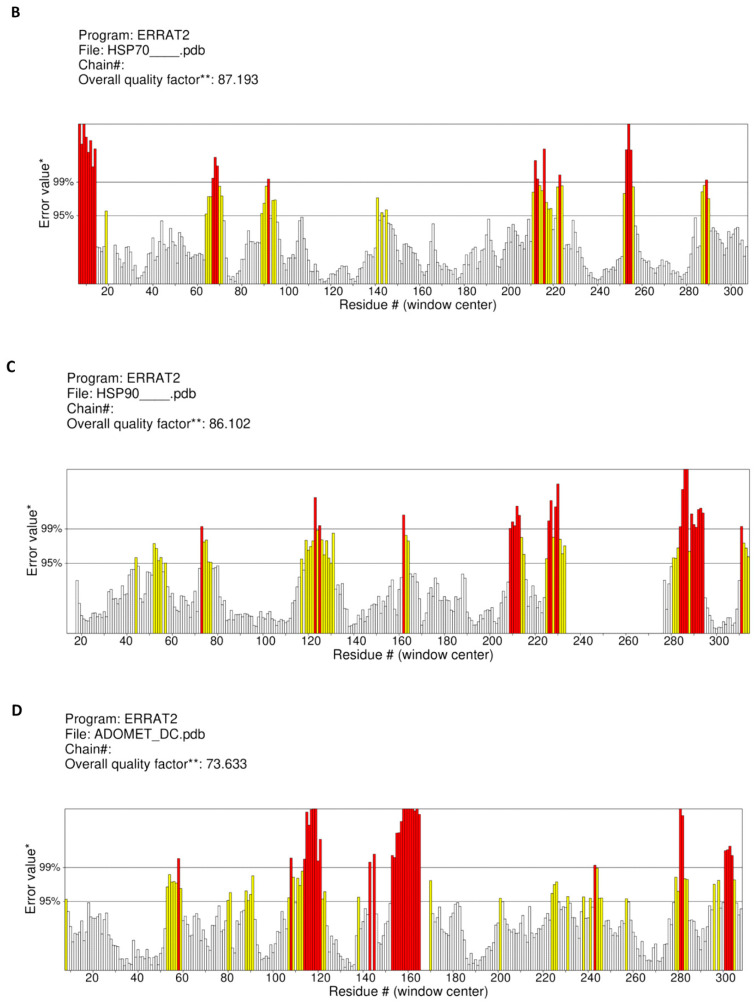

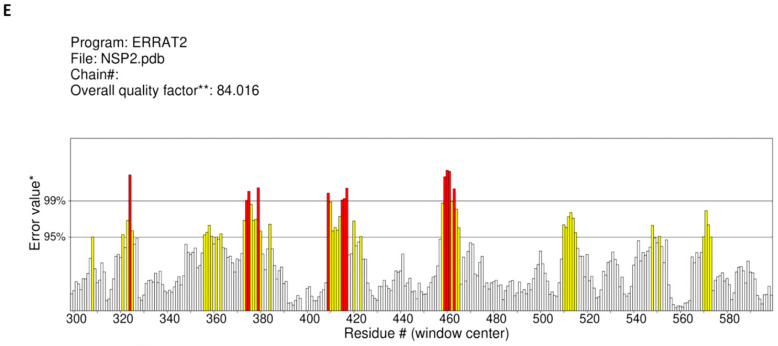
Representation of results obtained in validation through ERRAT for protein crystal structures of (**A**) HSP40, (**B**) HSP70, (**C**) HSP90, (**D**) AdoMetDC, and (**E**) NSP2. Red and yellow color represent the problematic part/less favored regeons while the white/grey color represents the normal part in the structure. #—number and * and **—have no meaning.

These structures were further validated, to be good quality models using ERRAT, with scores some above the … threshold (Figure 3). The percentage for their result were recorded as the overall quality factor. Red and yellow color represent the problematic part or unfavored regions while the white color represents the normal part in the structure.

### 3.3. Molecular Docking (Protein–Protein Interactions)

**Figure 4:**
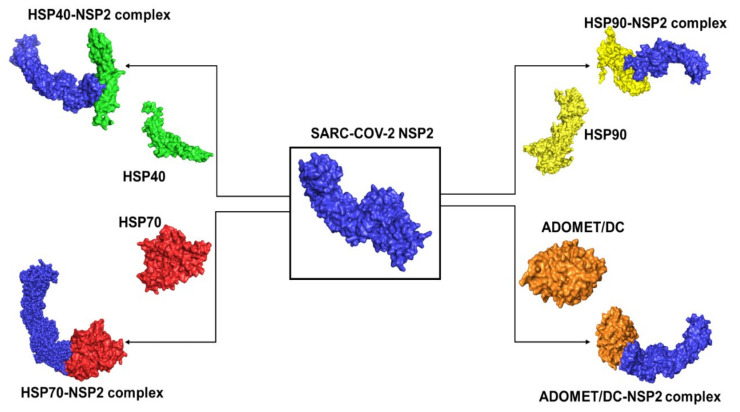
Diagrammatical representation of the best-result orientations of SARS-CoV-2 NSP2 (blue) bound to human heat shock proteins 40 (green), 70 (red), and 90 (yellow) as well as ADOMETDC (brown).

Protein docking was performed to investigate the association of the SARS-CoV-2 viral non-structural protein 2 (NSP2) with each of the major human heat shock proteins 40, 70, and 90 (Figure 4). ClusPro generates the best complexes, and the best results are selected based on PIPER interaction energies [24].

**Figure 5:**
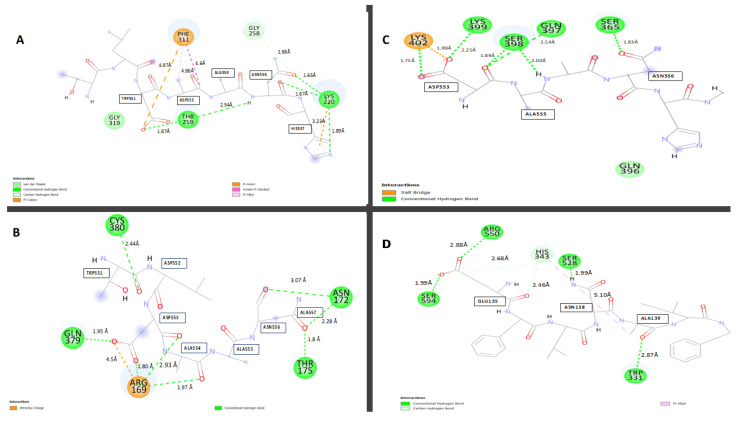
The 2D diagrams showing the interactions between (**A**) SARS-CoV-2 NSP2 and human HSP40, (**B**) SARS-CoV-2 NSP2 and human HSP70, (**C**) SARS-CoV-2 NSP2 and human HSP90, and (**D**) SARS-CoV-2 NSP2 and human AdoMetDC.

To find which amino acid residues are involved in the interactions between SARS-CoV-2 NSP2 and heat shock proteins 40, 70, and 90, their complexes were visualized and analyzed in Discovery Studio and the results are presented in 2D format (Figure 5).

The following protein docking algorithm was used to analyze these complexes. In protein–protein docking, intermolecular bonds—in particular hydrogen bonds—play a crucial role because they are typically used to determine whether two proteins are tightly or loosely bound to one another, without excluding the possibility of other bonds The algorithm states that the shorter the bond length, the stronger the interaction. Hydrogen bond length is measured by the distance from the donor atom to the acceptor atom [25,26]. In general, a hydrogen bond is considered optimal if the distance between the donor and acceptor atoms is less than the total of the acceptor atom’s atomic radius (1.5 Å), the hydrogen’s atomic radius (1.2 Å), and the bond length between the donor and hydrogen (1 Å). So, the longest hydrogen bonds are 3.5 Å and are desirable but not strong bonds. Anything longer than 3.5 Å is regarded as a poor dipole–dipole interaction. Good hydrogen bonds have a donor-to-acceptor distance of 2.8 Å, while some ultra-short hydrogen bonds have been observed with donor-to-acceptor lengths of 2 Å. Therefore, an ideal hydrogen bond is between the distance of 0.1 and 2.8 Å long [27].

### 3.4. Interaction between HSP40 and SARS-CoV-2 NSP2

The HSP40-NSP2 complex was examined and a total of 32 interactions were formed. The section with the highest interactions was chosen in a proportion of 1–10 amino acids and this was from amino acid Ala554 to His557 in NSP2 (Figure 5A). There were about eleven interactions in this region, which included six conventional hydrogen bonds and five carbon–hydrogen bonds. Some amino acids had numerous contacts, such as Lys220 from Hsp40, which had multiple contacts with His557 and Asn556, as well as forming interactions with different atoms from the same residue (Figure 5A).

Because of the multiple bonding, these amino acids are believed to serve a significant function in ensuring the interactions of the two proteins docked. The length of the hydrogen bonds varied between 1.89 Å and 2.68 Å for conventional hydrogen bonds and 2.89 and 4.97 Å for carbon–hydrogen bonds. The 2D diagram, shown above in Figure 5A, shows interactions between amino acids from the SARS-CoV-2 NSP2 protein with amino acids from human HSP40. The amino acid residues forming these bonds from SARS-CoV-2 NSP2 are amino acids from positions Trp551-His557, as shown in the figure below, the type of bond each residue forms are also shown in Figure 5A and Supplementary Table S1.

### 3.5. Interaction between HSP70 and SARS-CoV-2 NSP2

Docking heat shock protein 70 with NSP2 produced about 28 interactions with the most bonds from position 550 to 560 of NSP2 (Figure 5B). This region had eleven interactions. The amino acid residues responsible for this interaction were Asp553 which formed five bonds, interacting repeatedly with Arg169 and Gln379 from the Hsp70 (and Supplementary Table S2). The second amino acid residue that interacted with the HSP70 receptor protein was Asn556 which formed interactions with Asn172 and Thr175. Thr551 interacted with Cys380 of the receptor while Ala554 formed bonds with Arg169. Due to the multiple bonds formed by Arg169, it is worth highlighting that this residue might be very crucial in this interaction. This region had one electrostatic bond and ten conventional hydrogen bonds with varying distances. The electrostatic bond formed was 4.5 Å long and the conventional hydrogen bond lengths ranged from 1.80 Å to 3.09 Å. The HSP70-NSP2 complex predicted formed these interactions at the lowest interaction energy of −632.0 kcal/mol.

### 3.6. Interaction between HSP90 and SARS-CoV-2 NSP2

Human heat shock protein 90 (HSP90) docked with the SARS-CoV-2 viral NSP2 formed 19 interactions. Regarding the ligand–protein NSP2, Asp553 was the most crucial residue as it formed about six hydrogen bonds in the selected region (Figure 5C). The conventional hydrogen bonds observed had bond lengths that ranged from 2.74 Å to 2.54 Å and the one carbon–hydrogen bond was 2.71 Å long (and Supplementary Table S3). The length of these bonds fell within the ideal bond length, and due to this, it can be concluded that this complex is stable. The HSP90 functions primarily to complete the folding of very large proteins partially worked on by HSP70, and this may require strong binding; hence, this complex produced such stable bonds with binding energy of −710.5 kcal/mol.

### 3.7. Interaction between AdoMetDC and SARS-CoV-2 NSP2

The possible interactions between SARS-CoV-2 virus NSP2 and adenosyl methionine decarboxylase 1 was chosen as this enzyme plays a vital role in the regulation of the biosynthesis of polyamines, namely putrescine, spermidine, and spermine. A total of eleven interactions were observed at the chosen region between the amino acids 130–140 in NSP2 (Figure 5D). The ligand amino acid Glu135 formed five contacts with the receptor AdoMetDC with bond lengths ranging from 1.99 to 3.04 Å (and Supplementary Table S4). Of these, four were carbon–hydrogen bonds and one was a conventional hydrogen bond (Figure 5D). Interestingly, this amino acid, due to the multiple bonds it produces, may be very crucial for this interaction. The AdometDC-NSP2 complex formed these interactions at the lowest interaction energy of −610.29 kcal/mol.

### 3.8. Multiple Protein–Protein Docking

**Figure 6:**
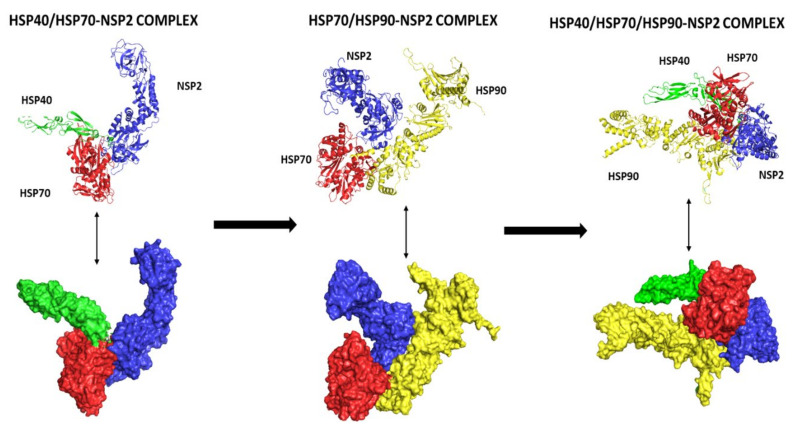
Multiple protein docking. The proteins were docked according to their functional activities and their functional partners.

**Figure 7:**
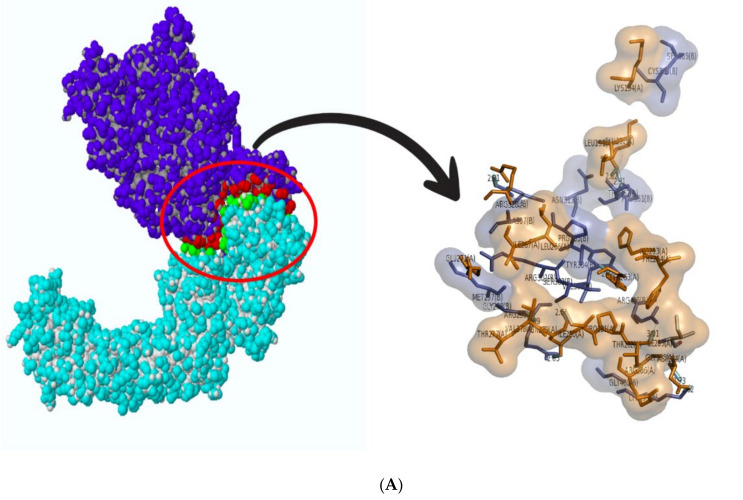

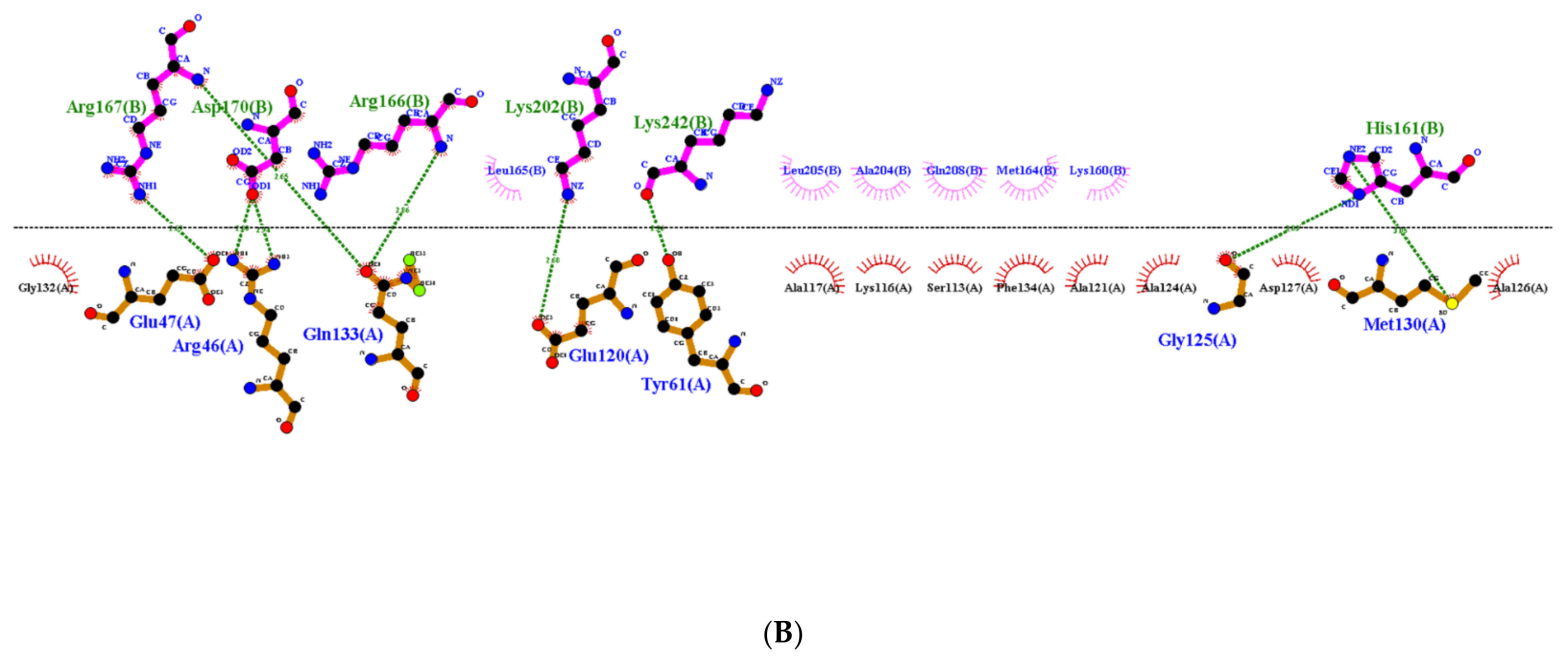
(**A**) Three-dimensional representation of HSP40/70 and NSP2 complex; (**B**) two-dimensional representation showing interaction between HSP40/70 and NSP2 complex generated using LigPlot++. Spiked semi-circles represent hydrophobic interactions, and hydrogen bonds are depicted by green dotted lines.

Multiple protein docking was performed to understand the interaction between Hsp 40, 70, and 90 with NSP2, and the resulting complexes are shown in Figure 6. The interactions were then analyzed using ligPlot++ (v1.4.2) to gain insights into the residues that are responsible for these interactions. The results for the HSP40/HSP70-NSP2 interactions are shown in Figure 7. A three-dimensional representation of the HSP40/70 and NSP2 complex is shown in Figure 7A. HSP40/70 complex is represented with green/red and NSP2 with blue color. Interacting residues are depicted in red for NSP2 and green for HSP40/70. A two-dimensional representation of the HSP40/70 and NSP2 complex is shown in Figure 7B. Chain A depicts HSP40/70, and NSP2 consists of chain B. Both hydrogen bonds and hydrophobic interactions participated in the HSP40/70 and NSP2 complex formation.

**Figure 8:**
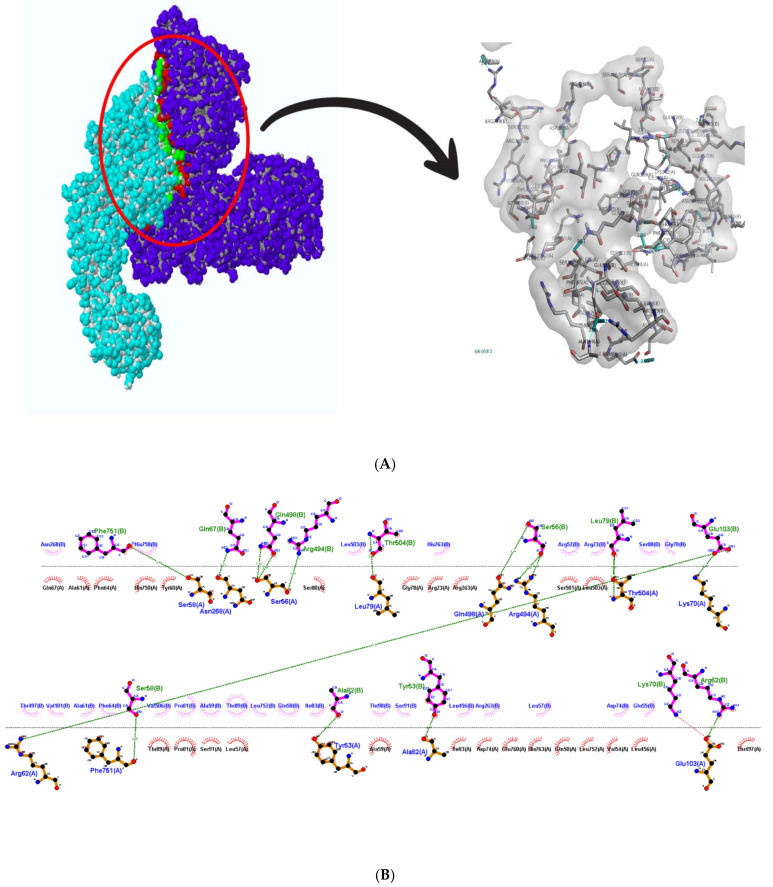
(**A**) Three-dimensional representation of HSP40/70/90 and NSP2 complex; (**B**) two-dimensional representation showing interaction between HSP40/70/90 and NSP2, a complex generated using LigPlot++. Spiked semi-circles represent hydrophobic interactions, and green dotted lines depict hydrogen bonds.

**Figure 9:**
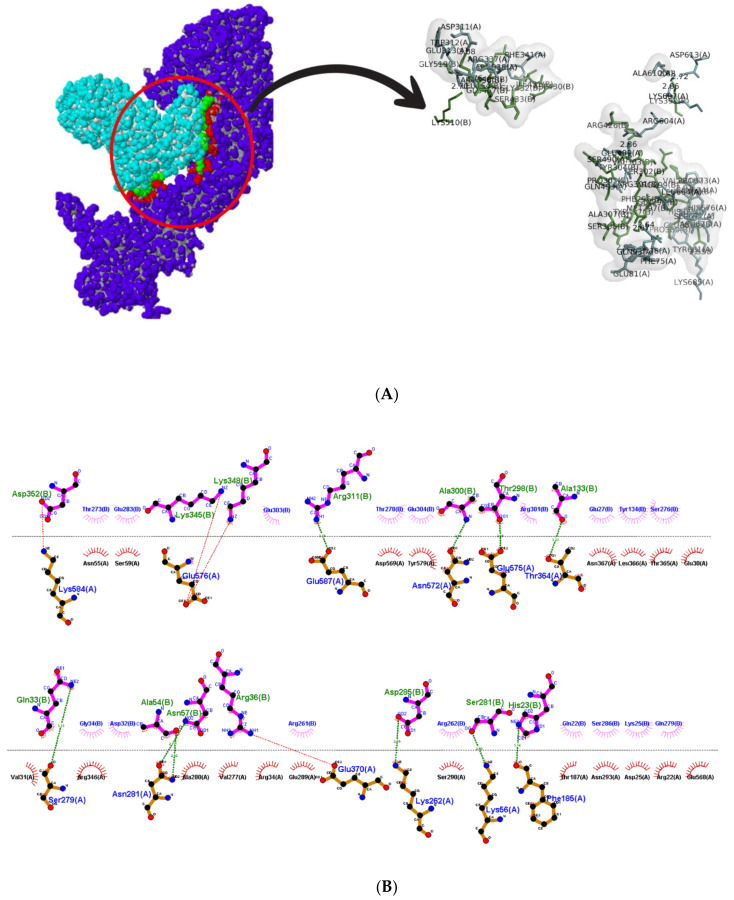
(**A**) Three-dimensional representation of HSP 70/90 and NSP2 complex; (**B**) two-dimensional showing interaction between HSP70/90 and NSP2, a complex representation generated using LigPlot++. Spiked semi-circles represent hydrophobic interactions, and green dotted lines depict hydrogen bonds.

This figure also reveals the bond types and bond length, and it can be concluded that these complexes formed good interactions. These were further put into molecular dynamics to further understand these interactions. Figure 8 depicts the interaction between HSP40/70/90 and NSP2 complex generated using LigPlot++ (v1.4.2). This figure depicts that NSP2 binds with these heat shock proteins using hydrogen bonds because many green dotted lines were observed. Figure 9 depicts HSP70/90 and NSP2 complex representation generated using LigPlot++ (v1.4.2). The spiked semi-circles represent hydrophobic interactions, and green dotted lines depict hydrogen bonds.

A three-dimensional representation of the HSP40/70/90 and NSP2 complex is shown in Figure 8A. The HSP40/70/90 complex is represented with green/blue/yellow colors and NSP2 with blue color. Interacting residues are depicted in red for NSP2 and green for HSP40/70/90. A two-dimensional representation of the HSP40/70/90 and NSP2 complex is shown in Figure 8B. Chain A depicts HSP40/70/90, and NSP2 consists of chain B. Both hydrogen bonds and hydrophobic interactions participated in HSP40/70/90 and NSP2 complex formation. Several residues in both protein chains contributed to the complex formation.

A three-dimensional representation of the HSP70/90 and NSP2 complex is shown in Figure 9A. The HSP70/90 complex is represented with red/yellow color and NSP2 with blue color. Interacting residues are depicted in red for NSP2 and green for HSP40/70/90. A two-dimensional representation of the HSP70/90 and NSP2 complex is shown in Figure 9B. Chain A depicts HSP70/90, and NSP2 consists of chain B. Both hydrogen bonds and hydrophobic interactions participated in HSP70/90 and NSP2 complex formation. Several residues in both protein chains contributed to complex formation.

### 3.9. Molecular Dynamics Simulations

#### 3.9.1. HSP 40, 70 and NSP2 Complex

**Figure 10:**
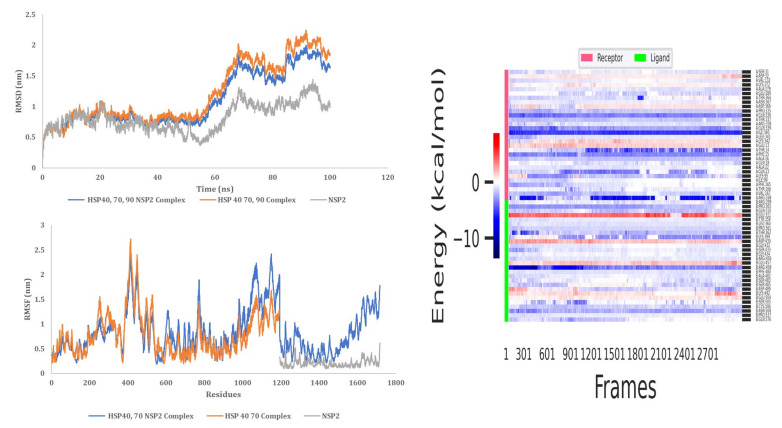
MD simulation analysis of HSP40/70 and NSP2 complex based on RMSD, RMSF, and decomposition energy analysis.

**Figure 11:**
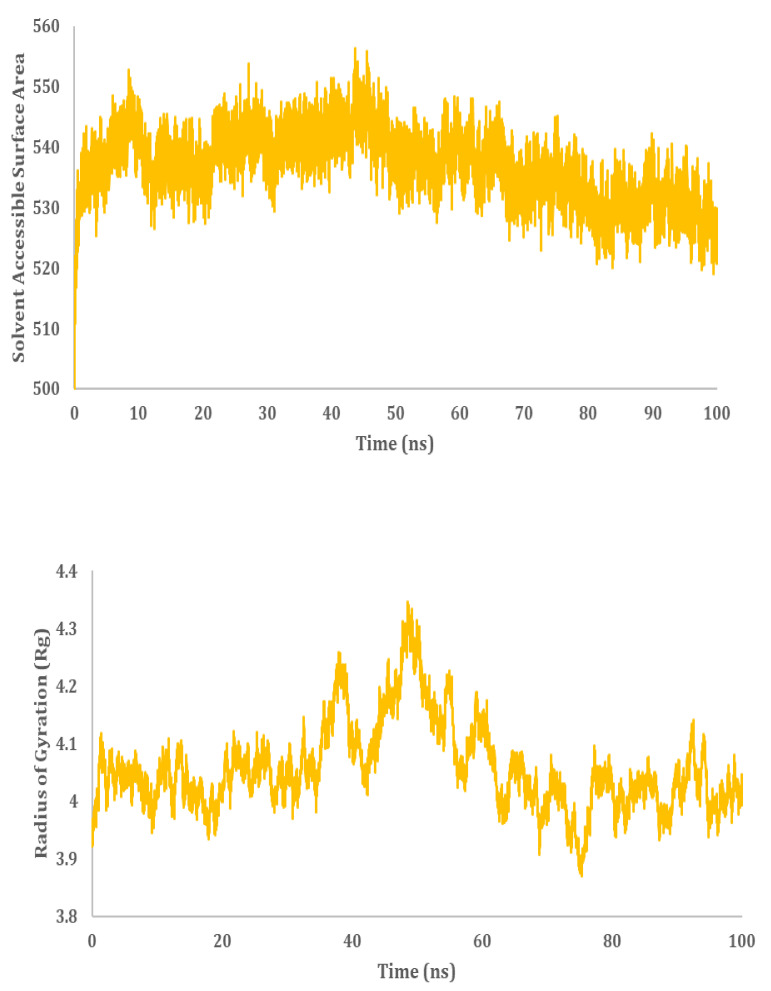
MD simulation analysis of HSP40/70 and NSP2 complex based on SASA and radius of gyration.

MD simulation analysis provided insights into the stability of the interaction between HSP40/70 and NSP2. RMSD analysis showed a stable interaction between HSP40/70 and NSP2 complex till 50 ns (Figure 10). The RMSD score exceeds 1 nm after 50 ns which is indicative of an unstable interaction. The RMSD score of unbound HSP40/70 complex exceeds 1 nm after 50 ns. The difference between the RMSF score of bound and unbound HSP40/70 is minimal. The RMSF score of unbound NSP2 is lower than bound NSP2. C-terminal residues of bound NSP2 showed maximum fluctuations. Decomposition energy analysis indicates energetically active residues that are important for complex formation. HSP40/70 residues Ile340 and Arg168 and NSP2 residues Arg458 and Asn508 are depicted to be active residues in HSP40/70 and NSP2 complex formation (Figure 10). SASA analysis indicated a decrease in surface availability after 40 ns. The radius of gyration indicated continuous expansion and compactness of the complex, and maximum fluctuations occurred from timestamp 40 ns to 60 ns (Figure 11). Overall analysis suggested an unstable interaction between HSP40/70 and NSP2, with a binding affinity of −6.5113907 kcal/mol (Supplementary Table S6). This is expected because HSP40 is not a strong binder. Hsp40 acts to deposit the substrate onto HSP70 and then detaches itself from the complex, thus leaving the complex slightly unstable for a short time.

#### 3.9.2. HSP40/70/90 and NSP2 Complex

**Figure 12:**
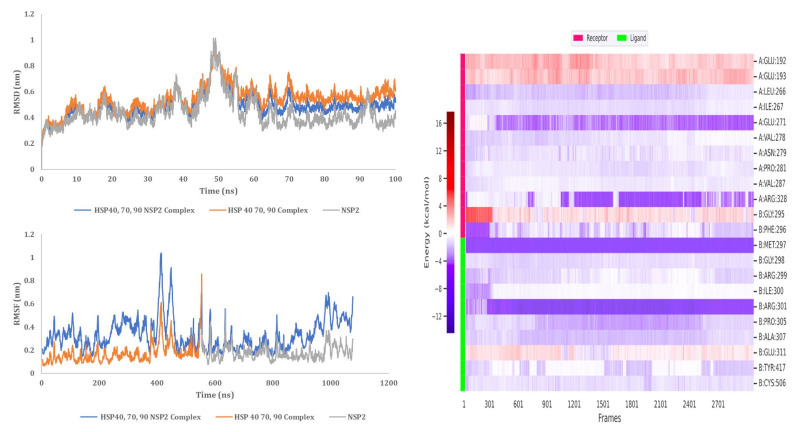
MD simulation analysis of HSP40/70/90 and NSP2 complex based on RMSD, RMSF, and decomposition energy analysis.

**Figure 13:**
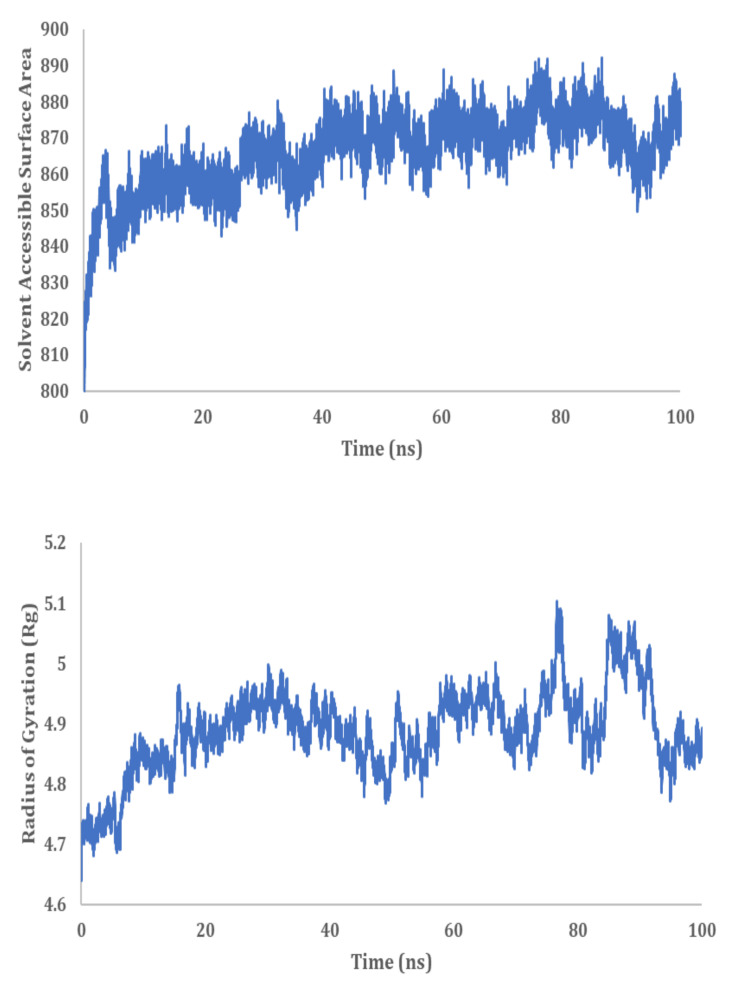
MD simulation analysis of HSP40/70/90 and NSP2 complex based on SASA and radius of gyration.

MD simulation analysis provided insights into the stability of the interaction between HSP40/70/90 and NSP2. RMSD analysis showed a stable interaction between HSP40/70/90 and NSP2. Throughout the 100 ns simulation, the RMSD score was below 1 nm (Figure 11). The RMSF difference between bound and unbound HSP40/70/90 and NSP2 indicated that N-terminal residues of NSP2 and C-terminal residues of HSP40/70/90 participate in the complex formation. Decomposition energy analysis indicates that HSP40/70/90 residues Glu271 and Arg328 and NSP2 residues Met297 and Arg301 are energetically active in HSP40/70/90 and NSP2 complex formation (Figure 12). The SASA analysis indicated a gradual increase in surface availability. The radius of gyration indicated expansion until 30 ns, followed by the compactness of the complex until 50 ns (Figure 13). After 70 ns, fluctuations in the complex radius indicate complex residues conformation change to attain the most suitable binding pose. Overall analysis suggests a stable interaction between the HSP40/70/90 and NSP2 complex, with a binding affinity of −8.7796222 kcal/mol (Supplementary Table S6).

#### 3.9.3. HSP 70 90 and NSP2 Complex

**Figure 14:**
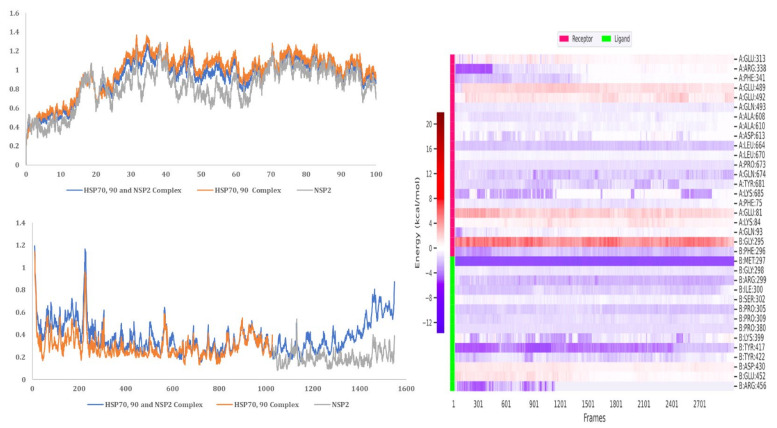
MD simulation analysis of HSP 70/90 and NSP2 complex based on RMSD, RMSF, and decomposition energy analysis.

**Figure 15:**
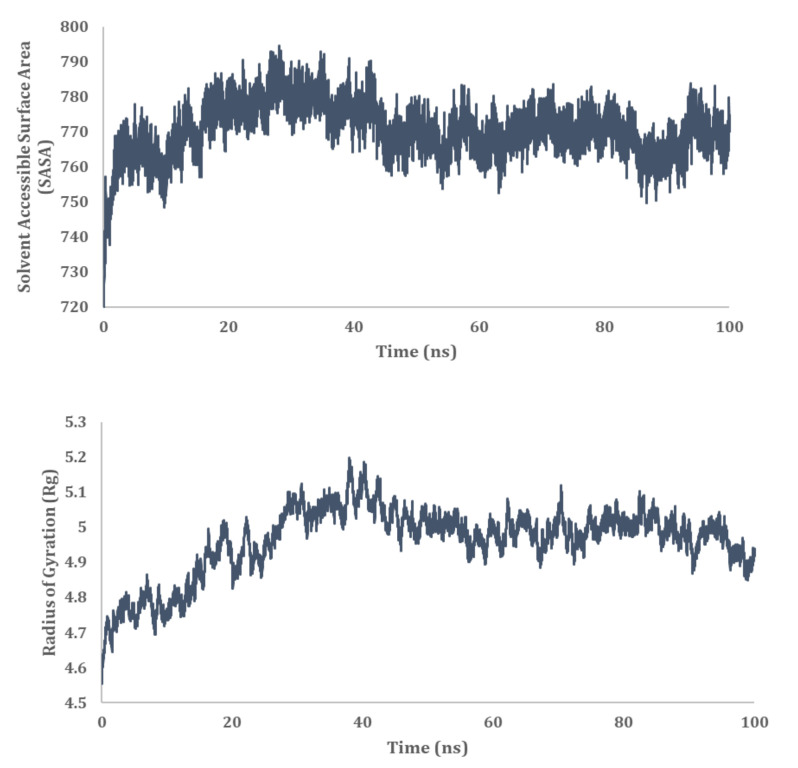
MD simulation analysis of HSP 70/90 and NSP2 complex based on SASA and radius of gyration.

MD simulation analysis provided insight into the interaction stability between HSP40/70/90 and NSP2. The RMSD analysis showed a stable interaction between HSP70/90 and NSP2 complex. The RMSD score exceeded 1 nm after 25 ns which indicated an unstable interaction (Figure 14). The difference between the RMSF of bound and unbound HSP70/90 is minimal. The RMSF score of unbound NSP2 is lower than bound NSP2. C-terminal residues of bound NSP2 showed maximum fluctuations. Decomposition energy analysis indicates energetically active residues that are important for complex formation. HSP70/90 residue Leu664 and NSP2 residues Met297 and Tyr417 are depicted as active residues in the HSP70/90 and NSP2 complex formation (Figure 14). The SASA analysis indicated an increase in surface availability until 30 ns which decreased from 30 ns to 50 ns. The radius of gyration also increased to 40 ns and fluctuated after the 40 ns timestamp (Figure 15). Overall analysis suggests a stable interaction between the HSP70/90 and NSP2 complex, with a binding affinity of −9.7906521 kcal/mol (Supplementary Table S6).

### 3.10. Pharmacophore Modeling

The pharmacophore modeling was performed to provide insight into whether the active amino acids identified by MD from the SARS-CoV-2 NSP2 can be inhibited from forming a complex with major HSPs. Residues Arg458, Asn508, Met297, Arg301, and Trp417 were screened against existing inhibitors through pharmacophore model generation. This step is crucial in computational drug discoveries, as this modeling conducts significant searches in the vast libraries of inhibitors made available on the computational platforms to choose molecules that are suitable candidates for the inhibition of certain activities of certain molecules. The pharmacophore modeling is based on an algorithm that demonstrates when two compounds share a similar spatial arrangement, common functionalities are also shared. This modeling provides an insight into a molecule’s properties that explain how it should react and the types of bonds it may form. The pharmacophore model does not concentrate on a single atom, but rather on chemical functionalities, making them useful tools for identifying similar activities between molecules, by using these models as a query [28].

**Figure 16:**
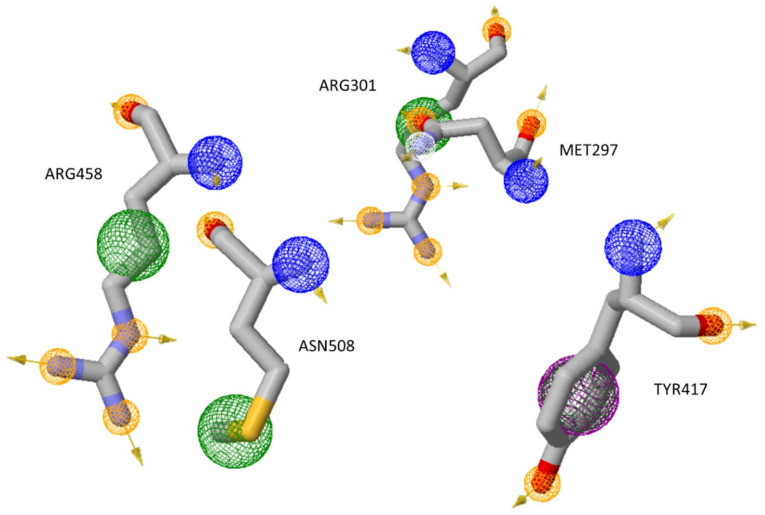
Structure-based pharmacophore features: yellow spheres denote the hydrophobic groups, and red and green spheres identify H-bond acceptors and donors. Red and blue spheres are negative and positive ionizable groups, respectively.

To generate a pharmacophore model, NSP2 amino acids Arg458, Asn508, Met297, Arg301, and Trp417 were selected because they were identified as the active residues in the formation of interactions. Pharmacophores were built on those residues important for the interaction between NSP2 and inhibitor-peptides (Figure 16). The yellow spheres represent the hydrophobic groups, and red and green arrows identify H-bond acceptors and donors, respectively. Red and blue spheres represent negative and positive ionizable groups, respectively. These active residues were then screened against existing zinc inhibitors. 

**Figure 17:**
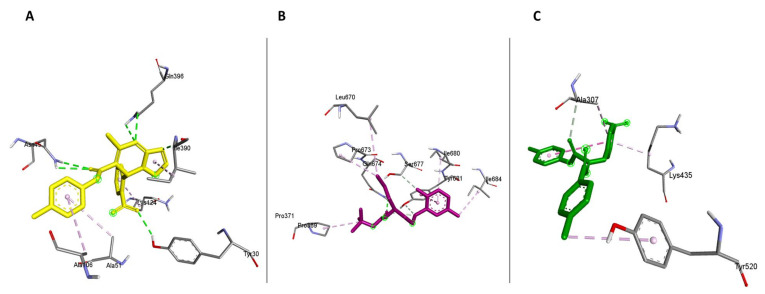
Molecular docking of the HSP40/70/90 complex with (**A**) ZINC01150525 [(1R, 3R)-3-(3, 3-dimethylmorpholin-4-yl)-1-(isopropyl amino) cyclopentyl] methanol, (**B**) ZINC395648 (3-Hydroxy-4-Methoxymandelate), (**C**) ZINC85324008 methyl-N-(4-methylphenyl) benzamide. The active amino acid residues for HSP40/70/90 complex are in a grey-like color. Dashed lines denote interactions (green for hydrogen bonds, pink for alkyl bonds, light pink for pi-alkyl bonds, and light green for conventional hydrogen bonds).

Each screening produced more than 10,000 hits but the top 10 (Supplementary Table S5) were regarded when each of the query features were submitted to ZincPharmer. The outcomes of potential inhibitors were selected by assessing the RMSD value and the number of bonds each outcome produced based on the Pharmer results. The top hits were [(1R,3R)-3-(3,3-dimethylmorpholin-4-yl)-1-(isopropyl amino) cyclopentyl] methanol, ZINC395648 (3-Hydroxy-4-Methoxymandelate), -methyl-N-(4-methylphenyl) benzamide). These were then docked onto the HSP complex using auto dock vina and results are shown in Figure 17. No molecular dynamics were performed for these.

Molecular docking shown in Figure 17 predicted ideal binding between the ligands mentioned above. Figure 17A had 5 hydrogen bonds and 4 alkyl bonds with binding energy of −674.16 kcal/mol and binding affinity of −5.6342 kcal/mol. Figure 17B depicted 4 hydrogen bonds together with 5 alkyl bonds with binding energy of −621.10 kcal/mol with binding affinity of −7.9426 kcal/mol. And Figure 17C predicted 2 hydrogen bonds and 4 alkyl bonds with binding energy of −453.77 kcal/mol and −4.6754 kcal/mol. There more bonds are produced between ligands and receptor the stronger the complex. The above complexes produce enough bonds to provide stable complex.

## 4. Discussion

This current study aimed to provide a clear understanding of the function of the SARS-CoV-2 non-structural proteins inside the human host. The sole primary goal of the SARS-CoV-2 virus when it infects a host is to multiply and make many copies of itself. It has been reported that the virus cannot initiate this process on its own. Instead, it presents its genome as an mRNA within the human cell, which the cell’s machinery for synthesizing proteins can read. This process results in the production of viral components necessary for the virus to grow and spread. Among these are molecules called non-structural proteins (NSPS) which it employs to ensure its development. In this study, NSP2 was selected. This protein functions mainly in weakening the host immune response, preventing it from attacking the virus when it is detected. Therefore, this study sought to analyze the functioning of (NSP2), and its association with human major heat shock proteins, toward finding potential inhibitors that may prevent these interactions. Among the processes hijacked by the SARS-CoV-2 virus is the function of heat shock proteins. In this study, the involvement of human heat shock proteins 40, 70, and 90 in the development of the SARS-CoV-2 virus was investigated.

The human heat shock protein 40 (HSP40) family are collectively known as co-chaperone molecules because they act as a first line of defense when a foreign substance invades the body. The HSP40 contains a J domain which it uses to activate the HSP70. Both HSP40 and HSP70 are typically localized in the cytosol which makes it much simpler for them to occasionally interact with one another during the protein modification process. The major function of HSP40 is to recruit substrates for HSP70, which it delivers to the HSP70 substrate-binding domain (SBD).

Since the HSP40 protein does not function in the modification process, it can be highlighted that strong binding between HSP40, and the substrate proteins is not expected. This was evident in this work since the HSP40-NSP2 complex had six weak interactions identified by long bonds at distances more than 2.8 Å, which is considered not an optimal distance for strong bonds. Such an interaction is regarded as a partially strong interaction. The predicted HSP40-NSP2 complex formed interactions at the lowest interaction energy of −709.54 kcal/mol.

HSP70 is made up of two major functional domains: the N-terminus NBD (nucleotide-binding domain) and the C-terminus SBD (substrate-binding domain) [29], each of which plays crucial roles in the Hsp70 function. The NBD supplies energy via ATP hydrolysis, while the SBD binds to the substrates and precedes the folding process. The folding process is quite a complex function and requires strong binding between the substrate protein and HSP70. In our study, a stable HSP70-NSP2 complex was established at the lowest interaction energy of −632.0 kcal/mol. This complex was deemed stable due to the short bonds formed with bond lengths ranging from 1.80 Å to 3.09 Å.

On special occasions, HSP90 comes into place to fold substrates that cannot be completely folded by HSP70, hence it was also wise to investigate the possible interactions between the SARS-CoV-2 NSP2 and HSP90. A stable receptor–substrate complex is also required since this protein must be tightly bound to its substrate. As expected, a stable complex between these two proteins was evident due to the short multiple bonds formed. The conventional hydrogen bonds were observed ranging from 1.75 Å to 2.54 Å and the carbon–hydrogen bonds had only one bond which was 2.71 Å long. The length of these bonds falls within the range of ideal bond length, and due to this, it can be concluded that this complex is stable requiring more validation by MD simulations.

AdoMetDC (S-adenosylmethionine decarboxylase) is a key regulator in the polyamine biosynthetic pathway because it creates the n-propylamine residue necessary to produce spermidine and spermine from putrescine. The possible interaction between human AdoMetDC and SARS-CoV-2 NSP2 was investigated, and the results revealed that the two proteins form very short bonds which implies a stable complex. The amino acid Glu135 from AdoMetDC formed five interactions with the receptor and distances ranging from 1.99 Å to 3.04 Å, and these had four carbon–hydrogen bonds and one conventional hydrogen bond. It is worth highlighting this amino acid due to the multiple bonds it produces, as it might be very crucial for this reason in the interaction of these two proteins. The AdoMetDC-NSP2 complex formed these interactions at the lowest interaction energy of −610.29 kcal/mol.

The molecular dynamic simulations provided a very good insight into understanding fully how the HSPs 40, 70, and 90 interact with NSP2, providing a clear indication of the residues that are largely active for this interaction. Decomposition energy analysis revealed energetically active residues that are important for complex formations. Analysis of the decomposition energy revealed Hsp40/70 Ile340 and Arg168 and the NSP2 residues Arg458 and Asn508 as energetically active residues that are critical for the formation of complexes. Moreover, MD simulation analysis provided evidence of the interaction stability between HSP40/70/90 and NSP2. We showed that HSP70/90 residue Leu664 and residues Met297 and Tyr417 of NSP2 played an active role in the HSP70/90-NSP2 complex while surface availability increased up to 30 ns and reduced from 30 ns to 50 ns, as per the SAS analysis. The RMSD analysis showed a stable interaction between HSP40/70/90 and NSP2. These amino acids play a major role in numerous cellular functions and are essential to cellular life. Arginine (Arg) is essential for wound healing, ammonia neutralization, and cell division [28]. Additionally, this amino acid possesses some therapeutic qualities, such as molecular signaling, which is critical for eradicating infections, regulating cytokine production, and mediating autoimmune diseases [30]. The growth and operation of the brain depends on the amino acid asparagine (Asn). For protein synthesis to occur during the replication of poxviruses, asparagine availability is also crucial [31]. According to Peng Li et al. (2007) [28], methionine (Met) inhibits the production of arginine. Tryptophan functions as a neurotransmitter and plays a role in preventing the generation of superoxide anion radicals and cytokines that are associated with inflammation. Tetrahydrobiopterin synthesis inhibitor is also known to act as an antioxidant in preventing the generation of superoxide and inflammatory cytokines [31].

To understand whether these active amino acid residues can be inhibited thus hindering the formation of complexes, these amino acids were screened against existing inhibitors through pharmacophore generation through ZINCPharmer, which then generated and aligned these with these proteins at their active areas. Figure provides an insight into how these inhibitors sit and interact in the active site [32].

We identified ZINC01150525 [(1R, 3R)-3-(3, 3-dimethylmorpholin-4-yl)-1-(isopropyl amino) cyclopentyl] methanol, ZINC395648 (3-Hydroxy-4-Methoxymandelate), and ZINC85324008 methyl-N-(4-methylphenyl) benzamide) as potential inhibitors. These were then docked using auto dock vina and results are shown in Figure 17 and no molecular dynamics were performed for these. According to [33] Benzamides have been found to have pharmaceutical properties against viral, bacterial, and fungal infections, tumors, and inflammatory and cancerous diseases [31]. 3-Hydroxy-4-methoxymandelate is a methoxy phenol organic compound and little is known about its medical properties. A study conducted by [34], on ZINC01150525 [(1R,3R)-3-(3,3-dimethylmorpholin-4-yl)-1-(isopropyl amino)cyclopentyl]methanol, was based on identification and preclinical pharmacology of ((1R,3S)-1-Amino-3-((S)-6-(2-methoxyphenethyl)-5,6,7,8-tetrahydronaphthalen-2-yl)cyclopentyl)methanol and discovered that this compound regulates a multitude of physiological processes including lymphocyte trafficking, cardiac function, vascular development, and inflammation [33]. Due to the medicinal inhibitory properties these molecules have, it is worth noting that they may play a huge role in the battle against SARS-CoV-2. Reena Kumari and colleagues carried out similar work in 2022, intending to identify or screen prospective antivirals for the PLpro of SARS-CoV-2. They found six compounds that possess these characteristics: 44560613, 136277567, S5652, SC75741, and S3833 [35].

## 5. Conclusions

The goal of the current study was to shed light on the behavior of the SARS-CoV-2 virus inside a human host. As is widely understood, viruses are unable to proliferate, replicate, or grow on their own. It was important to determine whether this virus uses the heat shock proteins and polyamines, which are known as housekeepers, when it infects a host. We showed that there is a high chance that this virus may come across these housekeeper proteins as they are all mainly found in the cytosol of the cell where the RNA of this virus is deposited during an invasion. Subsequently, we show stable interactions between HSPs and NSP2, and using MD simulations we verified that Arg458, Asn508, Met297, Arg301, and Trp417 were the amino acids that produced extremely stable interactions. These active amino acids were used to create a pharmacophore model. A pharmacophore model allows for the analysis of a single amino acid to obtain the types of bonds it produces and how it can be inhibited using the already existing inhibitors. When screened against existing inhibitors, the results revealed that the found molecules from the zinc database may be crucial inhibitors for the active amino acids as they perfectly aligned with the amino acids forming the active sites.

## Figures and Tables

**Table 1:** Introductory table containing information for human heat shock proteins HSP40, HSP70, HSP90, and the enzyme AdoMetDC retrieved on bioinformatic online databases.

Protein Name	Acc. Number	Source	Localization	Chromosome	Locus	Exons	Introns	Assembly ID
**Heat Shock Proteins**								
Heat shock protein 40 (HSP40)	O75953	Human	cytosol	9	NC_000009.12 (34,989,745.34998900)	5	4	GRCh38.p14 (GCF_000001405.40)
Heat shock protein 70 (HSP 70/HSPA4)	P34932	Human	cytosol	5	NC_000005.10 (133,052,013.133106449)	19	18	GRCh38.p14 (GCF_000001405.40)
Heat shock protein 90 (HSP 90)	P08238	Human	cytosol	6	NC_000006.12 (44,246,194.44253883)	12	11	GRCh38.p14 (GCF_000001405.40)
**Polyamine**								
Adenosylmethionine decarboxylase 1 (AdoMetDC)	AAH00171.1	Human	cytosol	6	NC_000006.12 (110,814,617.110895713)	6	5	GRCh38.p14 (GCF_000001405.40)
**Virus**								
SARS-CoV-2 replicase polyprotein	P0C6U8	SARS-CoV-2	Host cytosol	-	-	-	-	-

## Data Availability

The data utilized to substantiate the study’s conclusions are provided in the article.

## Supplementary Materials

The following supporting information can be downloaded at: https://www.mdpi.com/article/10.3390/cimb45120638/s1. Table S1: Tabular representation of the interactions between HSP40 and SARS-CoV-2 NSP2, showing amino acid residues from receptor and ligand-protein involved in the interaction, the types of bonds they produced, and the length of the bonds; Table S2: Tabular representation of the interactions between HSP70 (HSPA4) and SARS-CoV-2 NSP2, sowing amino acid residues from receptor and ligand-protein involved in the interaction, the types of bonds they produced, and the length of the bonds; Table S3: Tabular representation of the interactions between HSP90 and SARS-CoV-2 NSP2, showing amino acid residues from receptor and ligand-protein involved in the interaction, the types of bonds they produced, and the length of the bonds; Table S4: Tabular representation of the interactions between human AdoMetDC and SARS-CoV-2 NSP2, showing amino acid residues from receptor and ligand-protein involved in the interaction, the types of bonds they produced, and the length of the bonds; Table S5: ZincPharmer Top 10 results; Table S6: binding affinity for the studied complexes obtained from AREA AFFINITY.
