# Supplementary material for: In Silico Analysis of SARS-CoV-2 Non-Structural Proteins Reveals an Interaction with the Host’s Heat Shock Proteins That May Contribute to Viral Replications and Development
Source: Curr Issues Mol Biol. 2023 Dec 18;45(12):10225–47. doi: 10.3390/cimb45120638 (PMC10742040; doi:10.3390/cimb45120638)
Supplement: Supplementary file 1 [file cimb-45-00638-s001.zip › cimb-2659212-supplementary.pdf]

MOLECULAR DOCKING TABLES

Supplementary Table S1: Tabular representation of the interactions between HSP40 and SARS-CoV-2 NSP2, showing amino acid residues from receptor and ligand-protein involved in the interaction, the types of bonds they produced, and the length of the bonds.

PROTEIN	INTERACTION		BOND TYPE	BOND LENGTH (Å)
	**RECEPTOR**	**LIGAND**		
HSP40	LYS220: HZ1	HIS557: NE2	Conventional hydrogen bond	1.89
	LYS220: HZ2	ASN556: OD1	Conventional hydrogen bond	1.67
	LYS220: HZ3	ASN556:O	Conventional hydrogen bond	1.65
	THR259: HG1	ASP553: OD2	Conventional hydrogen bond	1.87
	ASN556:HN	THR259: OG1	Conventional hydrogen bond	2.54
	GLY258: HA1	ASN556: OD1	Conventional hydrogen bond	2.68
	THR259: HB	ASP553: OD2	Carbon hydrogen bond	2.89
	LYS220: NZ	HIS557	Carbon hydrogen bond	3.23
	ASP553: OD2	PHE311	Carbon hydrogen bond	4.97
	ALA554: C, O ALA555: N	PHE311	Carbon hydrogen bond	4.83
	PHE311-:	ALA554	Carbon hydrogen bond	4.6

Supplementary Table S2: Tabular representation of the interactions between HSP70 (HSPA4) and SARS-CoV-2 NSP2, sowing amino acid residues from receptor and ligand-protein involved in the interaction, the types of bonds they produced, and the length of the bonds.

PROTEIN	INTERACTICTIONs		BOND TYPE	BOND LENGTH (Å)
	**RECEPTOR**	**LIGAND**		
HSP70	ARG169:NE	ASP553: OD2	Electrostatic force	4.5
	ARG169: HH11	ASP553: OD1	Conventional hydrogen bond	1.8
	ARG169:HH21	ASP553: OD1	Conventional hydrogen bond	2.93
	ARG169: HH21	ASP553: O	Conventional hydrogen bond	1.97
	ARG169: HH21	ALA554: O	Conventional hydrogen bond	2.44
	ASN172: HD21	ASN556: OD1	Conventional hydrogen bond	2.28
	ASN172: HD21	ASN556: O	Conventional hydrogen bond	3.07
	ASN172: HD21	ASN556: O	Conventional hydrogen bond	3.09
	THR175: HG1	ASN556: OD1	Conventional hydrogen bond	1.80
	GLN379: HE22	ASP553: OD2	Conventional hydrogen bond	1.95
	CYS380: HG	THR551: O	Conventional hydrogen bond	2.44

Supplementary Table S3: Tabular representation of the interactions between HSP90 and SARS-CoV-2 NSP2, showing amino acid residues from receptor and ligand-protein involved in the interaction, the types of bonds they produced, and the length of the bonds.

PROTEIN NAME	INTERACTION		BOND TYPE	BOND LENGTH (Å)
	**RECEPTOR**	**LIGAND**		
HSP90	LYS402: HZ2	ASP553: OD2	Conventional hydrogen bond	1.75
	SER365: HG	ASN556: OD1	Conventional hydrogen bond	1.82
	GLN397: HN	ASP553: O	Conventional hydrogen bond	2.54
	SER398: HN	ASP553: O	Conventional hydrogen bond	2.00
	LYS399: HN	ASP553: OD2	Conventional hydrogen bond	2.25
	LY402: HZ1	ASP553: OD1	Conventional hydrogen bond	1.89
	ALA555: HN	SER398: OG	Conventional hydrogen bond	1.89
	GLN397: HA	ASP553: O	Carbon hydrogen bond	2.71

Supplementary Table S4: Tabular representation of the interactions between human AdoMetDC and SARS-CoV-2 NSP2, showing amino acid residues from receptor and ligand-protein involved in the interaction, the types of bonds they produced, and the length of the bonds.

PROTEIN NAME	INTERACTIONS		BOND TYPE	BOND LENGTH (Å)
	**RECEPTOR**	**LIGAND**		
	ASN138: HD21	SER528: OG	Conventional hydrogen bond	1.99
	ASN138: HD21	SER528: O	Conventional hydrogen bond	2.69
	TRP331: HN	ALA139: O	Conventional hydrogen bond	2.87
ADOMET-DC	ARG527: HH22	GLY144: O	Conventional hydrogen bond	1.76
	ARG550: HN	GLU135: OE1	Conventional hydrogen bond	2.86
	SER594: HG	GLU135: OE2	Carbon hydrogen bond	1.99
	TRP331: HD1	ALA139: O	Carbon hydrogen bond	2.28
	HIS343: HE1	GLU135: O	Carbon hydrogen bond	2.46

	SER528: HB2	GLU135: OE2	Carbon hydrogen bond	3.04
	SER594: HB1	GLU135: OE2	Carbon hydrogen bond	2.63
	HIS343	ALA139	Alkyl bond	5.10

Supplementary Table S5: ZincPharmer Top 10 results.

Name	RMSD	Mass	RBnds
ZINC01150525	0.509	464	13
ZINC85324008	0.511	331	10
ZINC395648	0.513	385	12
ZINC89721135	0.506	344	6
ZINC48717915	0.516	297	6
ZINC68063362	0.508	381	5
ZINC33813201	0.509	510	3
ZINC20193285	0.509	486	6
ZINC35568236	0.509	472	7

Supplementary Table S6: binding affinity for the studied complexes obtained from AREA AFFINITY.

Model name	Predicted binding affinity (log(K) kcal/mol
HSP 40/70 and NSP2	-6.5113907
HSP40/70/90 and NSP2	-8.7796222
HSP 70/90 and NSP2	-9.7906521
